# Function-Related Dynamics in Multi-Spanning Helical Membrane Proteins Revealed by Solution NMR

**DOI:** 10.3390/membranes11080604

**Published:** 2021-08-09

**Authors:** Koh Takeuchi, Yutaka Kofuku, Shunsuke Imai, Takumi Ueda, Yuji Tokunaga, Yuki Toyama, Yutaro Shiraishi, Ichio Shimada

**Affiliations:** 1 Cellular and Molecular Biotechnology Research Institute, National Institute of Advanced Industrial Science and Technology, Aomi, Koto, Tokyo 135-0064, Japan; tokunaga.y@aist.go.jp; 2 Graduate School of Pharmaceutical Sciences, The University of Tokyo, Hongo, Bunkyo, Tokyo 113-0033, Japan; happy@nmrlab.f.u-tokyo.ac.jp (Y.K.); ueda@nmrlab.f.u-tokyo.ac.jp (T.U.); yuki.toyama@utoronto.ca (Y.T.); 3 Center for Biosystems Dynamics Research, RIKEN, Suehiro, Tsurumi, Yokohama 230-0045, Japan; shunsuke.imai.ku@riken.jp (S.I.); shiraishi@nmrlab.f.u-tokyo.ac.jp (Y.S.)

**Keywords:** membrane protein, NMR, ion channel, GPCR, transporter, dynamics

## Abstract

A primary biological function of multi-spanning membrane proteins is to transfer information and/or materials through a membrane by changing their conformations. Therefore, particular dynamics of the membrane proteins are tightly associated with their function. The semi-atomic resolution dynamics information revealed by NMR is able to discriminate function-related dynamics from random fluctuations. This review will discuss several studies in which quantitative dynamics information by solution NMR has contributed to revealing the structural basis of the function of multi-spanning membrane proteins, such as ion channels, GPCRs, and transporters.

## 1. Introduction

Multi-spanning helical membrane proteins, such as GPCRs, ion channels, and transporters, play a critical role in transferring information and materials through a membrane. Since the function and malfunction of multi-spanning helical membrane proteins are critical in many biological processes and their associated diseases, understanding the functional mechanism of these proteins is of great interest from biological, medical, and pharmaceutical aspects. The recent advances in X-ray crystallography and cryo-electron microscopy (Cryo-EM) unveiled a significant number of structures of membrane proteins with biological, medical, and pharmacological importance. The number of published membrane protein structures is currently over 4000 and rapidly increases after the establishment of single-particle analysis of Cryo-EM.

**Figure 1:**
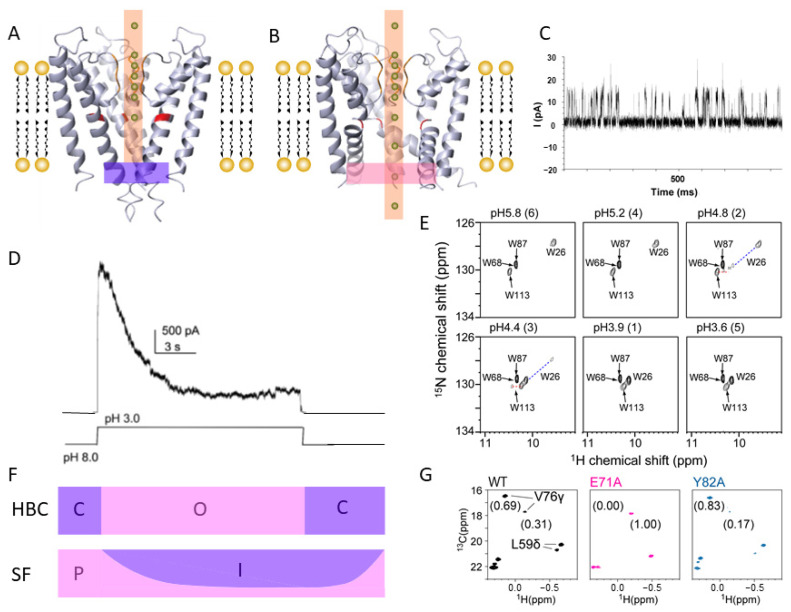
Functional dynamics of an ion channel. (**A**) Crystal structure of KcsA in the closed conformation at neutral pH (PDB ID: 1BL8). (**B**) Crystal structure of pore domain of MthK in the open conformation (PDB ID: 1LNQ). (**C**) Single-channel recording of KcsA in acidic condition [14]. (**D**) A macroscopic current of KcsA after pH jump. (**E**) Close-up view of the Trp indole region of the ^1^H–^15^N TROSY HSQC spectra of Hα-partially protonated/^2^H/^15^N-labeled Δ125–160 KcsA. The resonances from Trp-68 and Trp-87 overlap with each other. Assignments were established by site-specific mutagenesis. The dashed lines connect corresponding signals. (**F**) The conformational state of HBC and SF in the corresponding condition is shown in panel (**D**). (**G**) Methyl-TROSY spectra of WT (Left), the E71A (Middle), and Y82A (Right) mutants at pH 3.2 and 25 °C. The numbers in parentheses are the relative intensities of the two signals of V76 γ. The figure was reproduced from [14,15,16] with permission.

The atomic-resolution structures of membrane proteins have contributed to various research fields as a structural basis for understanding their functions. One of the early landmark examples is the determination of potassium ion channel structure by R. Mackinnon [1]. The structure of the KcsA potassium ion channel revealed the selective ion conductance mechanism of the channel by the formation of square antiprism coordination of carbonyl oxygens around each K^+^ binding site as if to mimic the waters of hydration [1,2] (Figure 1A). The following crystal structure of a calcium-dependent potassium channel MthK in the opened state unveiled the structural difference between the closed and opened potassium channels [3,4]. Under the closed conditions, the inner helix adopts straight conformation to form a bundle, closing the ion-conducting path in the intracellular side (Figure 1A). In contrast, the inner helices are in the bent configuration in the open conditions, which sprays out the intracellular gate, allowing the ion conductance (Figure 1B). In addition, the structures of ion channels with different regulation mechanisms have been solved to unveil how ion conductance is regulated by various inputs such as pH, ligands, temperature, membrane voltage, ion concentrations, mechanosensation, and protein–protein interactions, etc. [5,6,7,8,9,10,11,12]. The recent image processing technique in Cryo-EM even allows the classification of open and closed conformations of ion channels in the mixture to quantify the increased fraction of open conformation with the exposure to the inducers [13]. 

The dynamic behavior of membrane proteins is of importance as they are required to change their structure in response to various stimuli and transfer the information in and out of the membrane to exert their functions. For example, the single-channel recording shows that ion channels in the open conditions are not continuously permeating ions. They are instead under the equilibrium between open, closed, and inactive conformations, reflected in the stochastic profile of the ion permeabilization through a channel (Figure 1C). The open dwelling times are mostly in the order of milliseconds, which correspond to the exchange rate 1000 s^−1^. Since the open probability (i.e., the population of open state per unit time) determined from the open/closed dwell time and the ion conductivity in the open state defines the total ion flux, the activity of the ion channel can be described from the stochastic dynamic behavior of the proteins and is beyond the description by static snapshot structures.

NMR is an optimal spectroscopic technique to reveal such dynamics of proteins. NMR is able to define the function-associated dynamics in a site-specific manner and quantify the population of each conformation under an equilibrium, along with the rate of interconversion. For this purpose, various NMR measurements and stable-isotope labeling strategies are available. The quantitative analysis of the NMR relaxation rate can detect a high-energy functional state that populates less than one percent in an equilibrium. Due to its versatility to the condition, NMR can monitor the changes in structure and dynamics of membrane proteins associated with the functionally important parameters such as temperature, pH, ions, and inducers. The features would allow dissecting function-associated dynamics from random fluctuations. Thus, NMR would be of choice in understanding the functional mechanism of membrane proteins. In this review, we will overview some recent achievements that highlight the strength of solution-state NMR in the structure and dynamics analyses of the multi-spanning helical membrane proteins, such as ion channels, GPCRs, and transporters.

## 2. Ion Channels

As mentioned above, KcsA potassium channels are a hallmark example where structural studies, especially those from NMR, have contributed to understanding their functional mechanism. KcsA is a pH-dependent potassium channel derived from *Streptomyces lividans*. Structurally, KcsA is one of the simplest potassium channels consisting only of a pore domain harboring an ion-selective filter and an intracellular gate. Two-membrane spanning helices from each monomeric unit form a tetrameric assembly to form the ion conductance path at the rotation symmetry axis (Figure 1A). 

At neutral pH, KcsA adopts a closed state and is impermeable to K^+^ ions. As depicted by X-ray crystallography, inner transmembrane helices of KcsA form a bundle in the intracellular side to pinch the ion-conducting path. This intracellular helix bundle crossing (HBC) is a common structural feature among ion channels in closed conditions. While in the acidic condition, KcsA transiently and stochastically opens to conduct the K^+^ channel (Figure 1B). The microscopic current from patch-clamp multichannel recording showed that KcsA conducts transient maximum current right after reducing pH; however, the current decays exponentially to a certain inactivation level in 1–3 s [14] (Figure 1C). While the activation-coupled inactivation proceeds, the K^+^ conductance at the single-channel level remains invariant [17] (Figure 1D). Thus, the activation-coupled inactivation reflects the reduction of the open probability of the ion channel after activation. Indeed, the channels are reportedly open in a burst of activity before entering a long-lived nonconductive state [17]. The activation-coupled inactivation is present in almost all K^+^ channels and many other ion channels; thus, it reflects the common structural behavior of ion channels. 

While the open probability of KcsA in the inactivated condition is known to be as much as 10% [14], our NMR study revealed that intracellular HBC is fully open even in the inactivated condition [15]. In a series of ^1^H^15^N-TROSY HSQC spectra measured at various pH values, significant chemical shift changes were detected between pH 3.9 and 5.2, reflecting a conformational rearrangement associated with the gating of the ion channel. Since the 2D NMR measurement takes hours, the acidic condition spectra are those that experience the activation-coupled inactivation. The pH-dependent chemical shift changes were primarily observed in the residues near the intracellular HBC. Especially, the chemical shifts Trp-26 and Trp-113 are distinct at acidic and neutral pH, indicating that the intracellular gate changes to a completely different structure, presumably in open conformation at acidic conditions (Figure 1E). This observation supports the “dual-gate” properties of the K^+^ channel, in which the channel becomes permeable to K^+^ ions only when both intracellular HBC and the selective filter (SF) are in conductive conformation [15] (Figure 1F). Since the intracellular gate of KcsA is fully open even after a few hours of exposure to the acidic pH, the selectivity filter determines the channel’s open probability in the inactivated state. It should also be noted that from a pH-dependent measurement of KcsA, we revealed that the H25A mutation abolished this pH-dependent conformational rearrangement. These results indicate that the residue near the intracellular gate serves as a “pH-sensor” of the channel [15]. 

Further NMR investigation showed that the signals in the vicinity of the selectivity filter (SF) split into two peaks in acidic conditions [16] (Figure 1G). The relative intensity of the signal changes substantially with the temperature, which strongly suggests that SF in the acidic condition is under the equilibrium between two distinct conformations. Since the open probability of KcsA in the acidic inactivated condition is as much as 10% [17], we consider that the major peaks observed at 25 °C correspond to the impermeable conformation and the minor peak reflects the permeable conformation of the selective filter. From the analysis of the EXSY spectrum, the exchange rate between the two conformational states in SF was determined to be ~1 s^−1^. The value is closely in agreement with the inactivation kinetics of the channel [18]. 

To further verify this hypothesis, we compared the NMR spectra of the WT KcsA with those of the E71A and Y82A mutants (Figure 1G). The E71A mutant does not show inactivation. In contrast, the Y82A mutant experiences extensive activation-coupled inactivation, in which the open probability is less than 5%. In the NMR spectrum of the E71A mutant, the only signals that correspond to the permeable conformation (minor peak in WT KcsA) were mostly observed [16]. While in the Y82A mutant, the signal that corresponds to the permeable condition was much less intense than WT. These results support that major and minor signals observed in the WT KcsA are from impermeable and permeable conformational states of SF, respectively. 

Furthermore, the K^+^ titration experiments revealed that KcsA weakens the affinity to K^+^ ion in an acidic condition, and the loss of K^+^ ion at SF results in the deformation to an impermeable conformation [16]. Since NOE signals between the methyl group of Val-76 in SF and water were detected only in the impermeable state, we think that a water-binding instead of K^+^ ion leads to the blockage of the ion permeabilization at the SF. It should be noted that the reduction of K^+^ ion binding to the open state was also confirmed by recent solid-state NMR analysis of the constitutively open H25R/E118A mutant of KcsA embedded in a lipid bilayer at neutral pH [19]. 

As shown here, NMR successfully identifies the dual gate properties in KcsA, which thoroughly explains the electrophysiological profile of the channel. Independent NMR studies of KcsA by other groups also confirmed the structural and dynamic properties associated with the gating function of the ion channel [20]. It should also be noted that while the initial study was done in n-dodecyl β-d-maltoside (DDM) micelle, we also investigated the conformational equilibrium of KcsA in lipid bilayers [18]. Toward this end, reconstituted high-density lipoprotein (rHDL), also known as nanodisc, was used [21]. The rHDL is a disc-shaped particle of about 10 nm in diameter, in which a membrane scaffold protein with an amphiphilic helix structure holds the lipid bilayer structure inside. Since rHDL is monodisperse and soluble, it is suitable for NMR analysis. We reconstituted KcsA into rHDL and performed NMR observations. The NMR spectra in rHDL were similar to those in DDM, and there was no global structural difference between detergent-solubilized and rHDL-reconstituted KcsA. However, in the conformational equilibrium of the residues constituting SF, the ratio of impermeable state was higher in rHDL than DDM. This result strongly suggests that the lipid bilayer environment affects the structural equilibrium of KcsA and NMR is a suitable technique to dissect the mechanistic feature of ion channels embedded in lipid bilayer [18]. In line with this notion, it is shown that specific lipid composition strongly increases the KcsA open probability, indicating the critical role of the surrounding lipid bilayer in the gating of the KcsA channel [22]. 

Bax et al. reported the measurements of backbone ^15^N R_1_, R_1ρ_, ^15^N-(^1^H) NOE, and ^15^N CSA/dipolar cross-correlation for the KcsA channel in SDS micelles under conditions where this channel is in the closed state [23] as well as in the open state [24], and the dynamics at different time scales were successfully described. It should be noted that the study was conducted at a relatively high temperature (323 K) in SDS micelle. As a result, while the channel remains in the tetrameric form in the measurements under the closed condition, the channel dissociated into a monomeric form under the open condition at acidic pH, which might not be fully relevant to its functional state.

In addition, we recently investigated the effect of disease-related mutations on K^+^ channel conformational equilibrium [25]. Val-408 in the human Kv1.1 channel is located near intracellular HBC, and an Ala mutation at the position is known to associate with episodic ataxia. Mutational studies using the Shaker voltage-gated K^+^ (Kv) channel have revealed that increased sidechain volumes at the corresponding position substantially decrease the K^+^-permeability. Our NMR study revealed that the Ala to Val mutation at the corresponding residue (position 111) in KcsA stabilizes the closed conformation of HBC. The A111V mutant forms the structure closely resembling the closed state in the WT KcsA, even under the pH 3.0 condition. The results are consistent with the case of the Shaker Kv channel in that the bulky sidechain at the corresponding position results in a nonconductive phenotype. The Ala-111 contributes to extensive inter-helix van-der-Waals contacts in the open conformation, while the closed conformation is stabilized by introducing mutations with increased sidechain volumes. Thus, we considered that the stability of closed HBC conformation and disfavored sidechain van-der-Waals contacts in the open conformation would be the underlying mechanism of the increased population of the closed conformation in the KcsA mutants. Due to the structural similarity to KcsA, the disease-related mutations should also affect the gating of the human Kv1.1 channel.

We have also clarified the function-associated conformational equilibrium of the ATP-dependent ion channel, P2X [26]. In the α,β-methylene ATP-bound state, NMR signals from the residues in the transmembrane region and the residues that connect the ATP-binding site and the transmembrane region are in a conformational equilibrium between closed and open conformations [27,28]. NMR study also contributed to understanding the regulation mechanism of the NaK channel [29] and G protein-activated inwardly rectifying potassium channel [28,30], along with the structural characterization of ion channels [31,32,33,34,35] and their regulatory domains [36,37,38]. In addition, the inhibition mechanisms of ion channels by peptide toxins [39,40,41,42] were characterized by extensive structural and interaction analysis at residue-specific levels. These indicate the versatility of the NMR strategy to unveil the functional and inhibition mechanism of the class of multi-spanning helical membrane proteins.

## 3. G-Protein Coupled Receptors (GPCRs)

GPCRs are one of the largest families of membrane proteins in eukaryotes. In humans, there are more than 800 GPCRs, which function as receptors for neurotransmitters, hormones, and cytokines, etc. To date, more than 550 structures of >100 GPCRs have been deposited to PDB. Especially, the recent development of cryo-EM technology allowed us to determine the structure of GPCR in complex with heterotrimeric G proteins. While these structures substantially advanced the structural study of GPCRs, these structures are static and do not necessarily capture the major structures in physiological conditions. Instead, by using NMR and other biophysical methods, it becomes clear that GPCRs are under dynamic structural equilibrium with multiple conformations under physiological conditions to evoke multiple intracellular signaling pathways in various amplitudes. Especially, the development of in situ NMR strategy allows quantitative analyses of the dynamic structural equilibrium in GPCRs, such as the rate and amount ratio of exchange between different structures [43,44].

**Figure 2:**
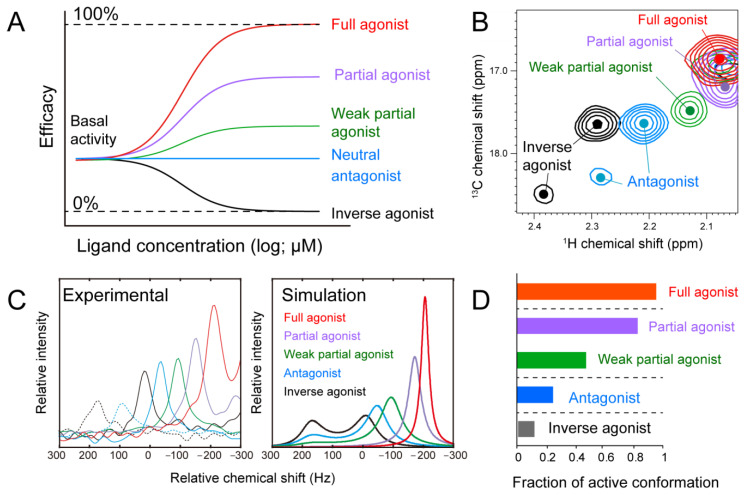
Efficacy of GPCR elucidated by NMR. (**A**) Pharmacological concepts of efficacy. (**B**) Overlay of the ^1^H–^13^C SOFAST-HMQC spectra of [α,β,β-^2^H_3_-, methyl-^13^C-Met]β2AR at 298 K in complex with an inverse agonist, carazolol (black), a neutral antagonist, alprenolol (cyan), a weak partial agonist, tulobuterol (green), a partial agonist, clenbuterol (violet), and a full agonist, formoterol (red). Only the regions with M82 resonances are shown. The signals are color-coded in the same way as in panel (**A**). (**C**) Experimental (left) and simulation (right) spectra of the ligand-dependent shift of the M82 resonances. Overlay of the ^1^H slice of M82 resonances in complexes with indicated ligands with different efficacy at 298 K is shown. (**D**) Conformational equilibrium quantified by NMR explains the efficacy of β2AR ligands. The fraction of active conformation that was obtained by fitting, shown in panel (**C**), was used. The figure was reproduced from [45] with permission.

One of the unique characteristics of GPCR is the presence of basal activity (Figure 2A). With the basal activity, GPCRs are always partially activated intracellular G proteins to a certain level. Upon the ligand binding, GPCRs both upregulate or downregulate the activation level compared to the basal level, depending on the ligand types. The degree to which the ligand activates the GPCR at the saturated concentration is called efficacy (Figure 2A). The ligands that inhibit the basal activity are called inverse agonists, and the ligands that do not change the basal activity are called neutral antagonists. A ligand that completely activates the target GPCR is called a full agonist. It is also known that there is a ligand called partial agonist that weakly activates the target GPCR. These differences in efficacy are known to affect the biological activity and usage of the drug. For the β2-adrenergic receptor (β2AR), it is reported that a full agonist offers a clinical advantage over a partial agonist in severe acute asthma. In contrast, partial agonists with fewer side effects are preferable in chronic treatment.

In the structure of the inverse agonist-bound β2AR, the fifth and sixth transmembrane helices (TM5 and TM6) interact with each other in the closed conformation. While, in the ternary complex of β2AR, full agonist, and G protein, the TM5 and TM6 of β2AR open outward, and the C-terminal helix of the α subunit of G protein enters the resulting cavity. The difference in the three-dimensional structures explains the mechanism by which a ligand induces the activation of GPCRs. However, the difference in the efficacy of each ligand and the basal activity of β2AR cannot be explained by a simple two-state conformational change from inactive to active conformations.

In order to unveil the structural mechanism underlying the difference in the efficacy of each ligand, we subjected β2AR for NMR analyses. For this purpose, NMR signals of Met-82, which undergoes a conformational change in conjunction with the P/I/F motif residues was used. The structure change in the P/I/F motif (Pro-211, Ile-121, and Phe-282 in β2AR) is known to trigger conformational changes in the intracellular side. In the inverse agonist-bound condition, two signals derived from Met-82 were observed [45] (Figure 2B). On the other hand, in the full agonist-bound condition, only one signal of Met-82 with a chemical shift distinct from that in the inverse agonist-bound form was observed [45] (Figure 2B). These observations indicate that the differences in the activation states of β2AR are reflected in the NMR spectra. 

The ^1^H chemical shifts of the Met methyl group are known to reflect the surrounding environment, especially the positions of the aromatic rings. In contrast, those of the ^13^C sidechain mostly reflect the χ^3^ dihedral angle of its own [46]. The ^1^H and ^13^C chemical shifts of Met-82 in the inverse agonist-bound state corresponded well to those in the crystal structure of β2AR. Similarly, the ^1^H and ^13^C chemical shifts of Met-82 in the full agonist-bound state correspond well to those of the full agonist-bound form. Therefore, the observed signals report the structural difference between inactive and active conformations of the GPCR [45]. It should be noted that two signals were observed for Met-82 in the spectrum, indicating that there are at least two distinct inactivation states in β2AR.

In the antagonist-bound state, two Met-82 derived signals are observed as it is similar to those in the inverse agonist-bound state; however, their chemical shifts are slightly different. A single Met-82 signal with a chemical shift between the inverse agonist- and full agonist-bound states was observed in the partial agonist-bound states. Additionally, depending on the strength in the activation level, the chemical shifts of the resultant spectra were changed; the weak antagonist is closer to that of the inverse agonist-bound state. Based on the correlation between the chemical shifts and the efficacy of the binding ligands, we concluded that β2AR is in equilibrium between the two inactive and one active conformation in each ligand-bound state and that the ratio of each state differs depending on the binding ligand (Figure 2C). In this case, the population of the active conformation upon the binding to the saturated concentration of ligands should reflect the efficacy. The ratio of the active conformation calculated from the chemical shifts of Met-82 signal in each ligand-bound state corresponds well to their G-protein activation capacity (Figure 2D) [45]. Similar function-related conformational equilibria of β2AR/β1AR in the transmembrane regions have later been investigated by other groups [47,48,49]. In addition, several groups investigated the conformational equilibria in the cytoplasmic regions by ^19^F-NMR, with chemical modifications by CF_3_ groups on the cysteine sidechains [50,51]. Interestingly, these reports suggest that the conformational equilibria in the transmembrane and cytoplasmic regions are only partially coupled [43].

The structure model of agonist-bound β2AR in solution was recently proposed by quantifying the paramagnetic relaxation enhancement (PRE) effect [52]. The structure of the agonist-bound β2AR in solution was different from the structure of any GPCR reported thus far. Compared to the structure of the inverse agonist-bound β2AR, TM6 is rotated about 90° clockwise when viewed from the intracellular side. In addition, the intracellular side of TM6 was closed as compared to the structure of the G protein-bound state. Due to the rotation of TM6, the G-protein-interacting residues on TM5 and TM6 cluster on the same surface to facilitate the interaction. Furthermore, the Leu mainchain amide signal broadening indicates the presence of a structural equilibrium in the transmembrane region of β2AR in the agonist-bound state, including the site where the effector binds. 

No such structural equilibrium was suggested in previous reports that utilize the extensive introduction of thermostable mutations. Thus, excessive mutation and modification of GPCR introduced to determine the structures by X-ray crystallography and Cryo-EM can be detrimental to the function-associated dynamics of proteins. It should also be noted that inserting fusion proteins in the effector-binding site also causes the loss of the function and associated dynamics of GPCRs. Thus, the observation of functional GPCR dynamics without modification would be critical in understanding the function of proteins with dynamic structural nature.

The measurements of backbone ^15^N relaxation rates are challenging for GPCR due to its molecular weight and limitation in deuteriation. Grzesiek et al. recently reported the ^15^N relaxation rates in the β1-adrenergic receptor in its *apo* form and seven ligand complexes [53], while the construct used retains extensive thermostabilizing mutations [48]. The measurements of ^19^F NMR relaxation times were also conducted for the ^19^F probes that are introduced to the functionally important sites [54,55]. The results clearly describe the changes in the amplitudes of local motions and the local reorientation frequency upon ligand bindings, which might reflect the exchange between conformational states associated with the GPCR [54,55]. A ^19^F CPMG relaxation dispersion experiment was conducted to quantitatively describe the rate of interconversion between the conformational states in β_2_-AR [51]. The strategy was also applied to obtain mechanistic insights into allosteric regulation of the A_2A_R by physiological cations [56] and ligand-dependent conformational equilibria in the transmembrane regions in β_1_AR [57]. It should also be noted that fast methyl dynamics were quantified in the presence of different ligands in A_2A_R by using triple-quantum relaxation data [58], as well as in sensory rhodopsin II [59] and bacteriorhodopsin [60]. 

The NMR analyses of GPCRs were also conducted in a lipid bilayer environment using rHDL. Since GPCR embedded in rHDL is equivalent to a protein with a molecular weight of 200 K, the NMR spectrum of fully protonated β2AR-rHDL is rather poor. Thus, the amino acid selective deuteration was employed for the residues located in the proximity of the observing methionine residues to enhance the methyl TROSY effect. The procedure enhances the sensitivity of the NMR signal by ~5 fold [61]. Similar to that observed in DDM micelle, the NMR spectra of β2AR-rHDL bound to various ligands reflected the distinct efficacy of each ligand. However, the chemical shift of the β2AR in the partial agonist-bound state was closer to that of the full agonist-bound state in rHDL than those in micelles, indicating that the proportion of the active form was slightly higher in the lipid bilayer environment [61]. In addition, the signal in the partial agonist-bound state was significantly broader than that in micelles, and two separated signals were observed in the weak partial agonist-bound state. These results indicate that the exchange rate between the active and inactive forms is slower in the lipid bilayer environment than in micelles. 

Although the GPCR dynamics study conducted in a lipid bilayer environment is limited, one more example was recently presented for the leukotriene B4 receptor BLT2 [62]. It is shown that BLT2 explores at least four different conformational states for the unliganded condition, and the relative population of the conformational states is modulated by ligands and the sterol content of the membrane.

We also calculated the G-protein activation capacity using the percentage of active β2AR in micelles and rHDL derived from NMR signals and compared it with the experimental G-protein activation capacity in β2AR-expressing cells. The results showed that the G-protein activation in rHDL corresponded better to the experimental values in cells than in micelles. Therefore, the lipid bilayer environment has an essential effect on the activity of β2AR. From the exchange rate of the structural equilibrium, we can also estimate the time required to activate the downstream signal. The results showed that the activation of β2AR occurred in ~1 ms, followed by the activation of G protein in ~100 ms, and the change in intracellular cAMP concentration in ~1 s. This estimation closely matches the experimentally measured time required for the G protein activation and the change of the intracellular cAMP concentration. The rapid activation of GPCRs is thought to be advantageous for neurotransmission and sensory reception.

The NMR observation of critical methionine residues also explained the structural basis for the selective activation of intracellular signaling. GPCRs not only activate G-protein signaling but also evoke G-protein-independent arrestin signaling after phosphorylation of the intracellular region by GPCR kinases (GRKs). GPCR ligands promote differing degrees of signaling in the G-protein and arrestin pathways called biased signaling [63,64]. Ligands that promote both signaling pathways are called balanced ligands, whereas those that activate one of the signaling pathways are called biased ligands. Although the crystal structure of the rhodopsin–arrestin complex is available, the structure of arrestin-bound rhodopsin was almost identical to that of G protein–mimetic peptide bound form [65]. Thus, the structural basis of biased signaling is largely unknown.

In μ opioid receptor (μOR), Met-245, adjacent to the aforementioned P/I/F motif, showed distinct chemical shifts in the activated form, depending on the signaling bias conditions [66]. The signal corresponding to the activated conformation of the G-protein-biased-partial-agonist (TRV130) bound form, and that of the β-arrestin-biased mutant bound to full-agonist shifted in the opposite direction relative to that of the balanced-full-agonist bound state. In addition, the chemical shifts of Met-245 correlated well with the bias factors, which is the ratio of the β-arrestin signaling efficacy to the G-protein signaling efficacy in each state. These results indicate that μOR is under an equilibrium among distinct activation states that favor the G protein activation and arrestin activation. The G protein-biased ligands induce the activation state that is suitable for G protein signaling, while the arrestin-biased mutant populates more to the activation state that favors the initialization of arrestin signaling. The chemical shifts of Met-163, Met-257, and Met-283, located in the intracellular side of the TM3, TM5, and TM6, respectively, also showed the chemical shift change that reflects the signaling bias. These residues are located in positions that reflect TM3, TM5, TM6, and TM7 conformations. Thus, the conformational equilibrium described above accompanies the coupled conformational changes in these TM helices [66]. The intracellular cavity, which is composed of TM3, TM5, TM6, and TM7, seems to exist in equilibrium between multiple open conformations, including the conformations that preferentially activate either G-protein-mediated signaling or β-arrestin-mediated signaling. Each biased ligand seems to shift the equilibrium between the multiple open conformations toward the conformation that preferentially activates G-protein-mediated signaling or arrestin signaling. The chemical shift derived from the NMR experiment thus provides information to dissect or verify the enhanced selectivity of a ligand to a certain signaling pathway [66]. 

As described above, phosphorylation of the C-terminal region of GPCRs by GRK initializes the arrestin signaling. However, the structure of GRK-phosphorylated GPCRs has not been described in detail. It is known that the lipid bilayer environment is important for the phosphorylation of GPCRs by GRK; thus, we reconstituted the β2AR reconstituted in the rHDL to obtain phosphorylated β2AR. The NMR spectra of the phosphorylated β2AR-rHDL showed that the signal from Met-215, located in TM5, underwent a significant chemical shift change upon phosphorylation. In addition, the Met-279 signal originating from the TM6 became broader. Therefore, phosphorylation of the C-terminal region affects the structure of the transmembrane region of β2AR. In addition, the Met-215 signal in the unligated phosphorylated β2AR was observed between those of unphosphorylated and arrestin-bound phosphorylated states. Thus, the phosphorylated β2AR partly forms a structure that is similar to the arrestin-bound structure [67].

Furthermore, by utilizing the segmental labeling strategy using the trans-splicing technique, the NMR signals originating from the C-terminal region were selectively detected [67]. The Hα and ^13^Cα chemical shifts of the C-terminal region of the full agonist-bound state of β2AR reconstituted in the rHDL indicated that the region does not form a specific secondary structure. Upon the phosphorylation by GRK, part of the signals from the C-terminal region were significantly broadened. In particular, the signal originating from the γ2 methyl group of Thr-360 was broadened toward the ^13^C axis, suggesting the existence of the equilibrium between multiple rotameric states with different χ1 angles on a timescale of μs–ms. In addition, the saturation transfer from the transmembrane region of β2AR to the C-terminal residue, Thr-360, was observed. Therefore, the corresponding region is close to the transmembrane region upon phosphorylation. Since arrestin recognizes both the transmembrane region and the phosphorylated C-terminal region of the GPCR, the proximity of the phosphate group to the transmembrane region would be required to form the surface suitable for arrestin interaction. Since the intracellular side of the transmembrane region of GPCR, including β2AR, is rich in basic residues, these basic residues would contribute to the interaction to the phosphorylated and negatively charged C-terminal region. The interaction analyses of phosphorylated GPCR and arrestin by NMR have also revealed that the distinct receptor phospho-barcodes are translated to specific arrestin conformations and define selective signaling [68].

As described above, NMR revealed the dynamic structural equilibrium of GPCRs in the lipid bilayer environment close to physiological conditions, which is directly related to their activity. The applications of NMR to understand the dynamics of GPCRs are extensively pursued as illustrated in recent reviews [43,69,70,71]. It should also be noted that the dynamics of the human neuropeptide Y receptor type 2 reconstituted into dimyristoylphosphatidylcholine (DMPC) membrane were recently investigated by solid-state NMR spectroscopy along with molecular dynamics simulations [71,72]. From ^15^N and ^2^H NMR spectra and the ^13^C MAS NMR spectra, a large amplitude of structural fluctuations in a liquid-crystalline membrane was described at physiological temperature, which further indicates the high mobility of the protein in the lipid bilayer membrane. The information would also guide the ligand design to control the efficacy and signal selectivity, distinct from the conventional ligand design that uses the only affinity to the target protein as an indicator.

## 4. Transporters

Exchanging substances across the membrane to supply nutrients and remove waste products is fundamental for cells to keep biological activities. The exchanges are carried out by membrane proteins such as channels and transporters in a lipid bilayer. While channels passively transport ions through the pore structure, most transporters actively transport their ligands using energy molecules, such as ATP and/or concentration difference of protons, ions, and other substances across the membrane. Since transporters are also important to uptake or excrete drug molecules, they are critically involved in the pharmacokinetics and therapeutic efficacy of drugs. One of the hallmark examples is multidrug efflux transporters, which are also recognized as the most fundamental biological defense mechanism widely distributed in all three kingdoms of life. For the sake of clarity, here we focus on the structural study of multidrug efflux transporters. 

There are two distinct types in multidrug efflux transporters; one is the ABC (ATP-binding cassette)-type, which utilizes the energy derived from the hydrolysis of ATP molecules for transport activity. The others are ATP-independent transporters, mostly antiporters, which carry in H+/Na^+^ ion and drugs in opposite directions. Recent advances in the structural analysis have revealed how transporters achieve active transport through various mechanisms and work as sophisticated molecular machines. As for P-glycoprotein (P-gp), an ABC-type multidrug efflux transporter, X-ray, and Cryo-EM structures unveiled overall structural architectures, as well as the ligand and inhibitor recognition mechanism of the transporter [73,74,75,76]. Most of the P-gp structures are inwardly facing, presumably because the outward-facing state is not stable enough to be crystallized. Recently, the crystal structure of *Cyanidioschyzon merolae*, red algae, P-gp was determined in an outward-facing conformation with bound nucleotide, representing the first case that both inward and outward-facing conformations are determined for the eukaryotic homolog [77]. As for antiporter, the AcrAB has been extensively analyzed to unveil how trimeric AcrB transporter recognizes and eliminates various drugs in a functionally rotating mechanism based on the detailed three-dimensional structures [78,79]. Even a whole architecture of the AcrAB–TolC multidrug efflux pump that is composed of the outer-membrane channel TolC, the secondary transporter AcrB located in the inner membrane, and the periplasmic AcrA, which bridges these two integral membrane proteins, were experimentally visualized by Cryo-EM [79,80]. 

**Figure 3:**
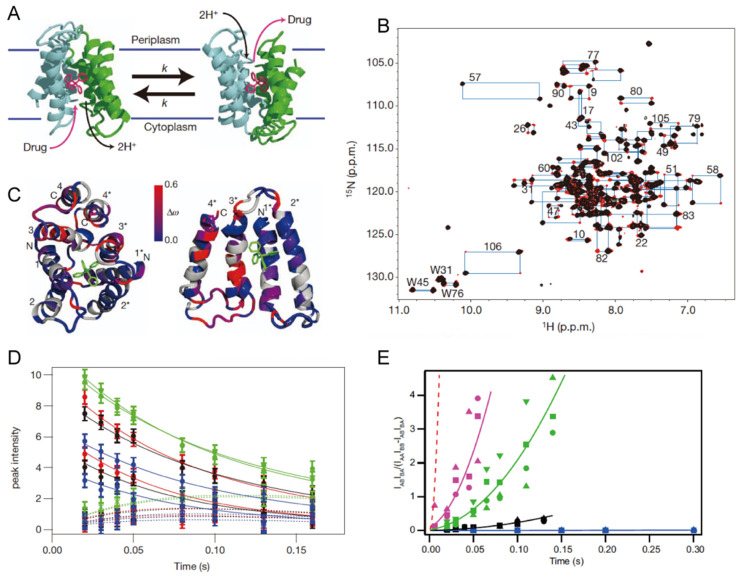
Transportation coupled conformational exchange of EmrE transporter revealed by NMR. (**A**) Proposed transport mechanism of EmrE by antiparallel asymmetrical dimer. The two conformational states adopt an identical structure in opposite orientations with respect to the membrane. Interconversion between the inward-open and outward-open conformations enables EmrE to transport its substrate. (**B**) Overlay of conventional ^1^H–^15^N TROSY HSQC spectrum (black) with the TROSY ZZ-exchange spectrum (red, 100 ms mixing time). Blue lines connect exchange peaks and auto-peaks, and residue numbers are indicated. (**C**) Mapping of normalized chemical shift changes (Δω) between auto-peaks in the structure of EmrE. (**D**) The global fit of auto- (solid circles) and cross-peak (open circles) intensities in the ZZ-exchange experiment. (**E**) Composite peak ratio analysis of ZZ-exchange data. Significantly different exchange rates for EmrE bound to TPP^+^ (black) and the other slow-exchanging derivatives, EtTPP^+^ (magenta), MBTPP^+^ (green), and DPhTPP^+^ (blue). Global fits are depicted with solid lines, along with the experimental data points. The dashed red line corresponds to a simulation of the composite peak ratio of fast-exchanging MeTPP^+^ using the rate of conformational interconversion determined via line shape analysis. This figure was adapted from [86] with permission.

EmrE is another multidrug efflux transporter that is extensively analyzed by multiple structural methods. EmrE is a 110-amino-acid residue antiporter that belongs to the small multidrug resistance (SMR) transporter family. The protein exports a broad range of cationic aromatic substrates in exchange for two protons in the opposite direction [81]. It is known that the initial structure of EmrE was erroneously determined due to an improper process in the in-house program [82,83]. The current consensus topology of the EmrE structure is an asymmetric dimer with antiparallel orientation for the monomers, which is consistent with initially determined by two-dimensional crystal cryoelectron microscopy (cryo-EM) [84], the revised X-ray structure [85], and NMR as discussed below [86] (Figure 3A). In the ^1^H–^15^N transverse relaxation-optimized spectroscopy (TROSY) spectrum of TPP^+^-bound ^2^H/^15^N-EmrE embedded in isotropic bicelles, twice as many peaks as expected for a monomer of EmrE with almost equal intensity was observed [86] (Figure 3B). Two possibilities are consistent with the NMR data: (1) parallel, symmetric EmrE dimers interconverting between inward- and outward-facing states and (2) the unique model of asymmetric antiparallel EmrE dimers interconverting between two states that are identical but open to opposite sides of the membrane. With equal populations, asymmetric antiparallel EmrE dimers can be expected, as symmetric parallel EmrE dimer model requires two distinct conformations with exactly equal free energies. In contrast, asymmetric antiparallel EmrE inherently shows equal populations because each dimer consists of one monomer in each conformation. This expectation was further confirmed by bulk and single-molecule FRET and cross-linking experiments [86]. 

TROSY-selected ZZ-exchange experiments were performed to quantify the interconversion between the two states. As a result, nearly every resolved TROSY peak was assigned to exchange pairs, suggesting a widespread conformational exchange in the EmrE dimer (Figure 3C). The kinetics of conformational exchange was determined by varying the mixing time and fitting the cross-peak build-up and auto-peak decay as a function of time (Figure 3D). For TPP^+^-bound EmrE in isotropic bicelles at 45 °C, a global conformational exchange with a rate constant of 4.8 ± 0.5 s^−1^ was defined, consistent with the previously observed rate determined by fluorescent measurements. A similar experiment conducted for various ligands showed that the rate of interconversion between the inward- and outward-facing states of EmrE varies significantly depending on the substrate [87] (Figure 3E). Thus, the bound substrate seems to control the rate of this critical step in the transport process. In addition, a cross-link that dramatically reduces the conformational exchange of EmrE impairs ethidium efflux activity. The observation directly confirms that the conformational exchange is vital for substrate transport [88]. Very recently, the structure of drug-bound EmrE in phospholipid bilayers was determined by 214 protein–substrate distances originating from ^19^F and ^1^H solid-state NMR experiments using a fluorinated substrate, tetra(4-fluorophenyl) phosphonium (F4-TPP^+^). The structure showed that F4-TPP^+^ lies closer to the proton-binding residue E14 in one subunit than the other subunit in the dimer, explaining the asymmetric protonation [89]. This structure and dynamics information of EmrE obtained by NMR would contribute to the future design of inhibitors for the multidrug resistance systems.

## 5. Conclusions and Future Perspectives

As described here, the conformational dynamics in multi-spanning helical membrane proteins quantitatively define their activity. Membrane proteins are under conformational equilibrium between different conformational states that are tightly associated with distinctive functional states. The relative population of the different conformational states can quantitatively predict the functionality of the membrane protein under certain conditions. The exchange rates between the distinct conformational states described by NMR can ultimately define the time scale of the cell response as exemplified for ion channels and GPCRs. Since NMR can provide various methods that allow detecting and quantifying protein dynamics on various timescales at a semi-atomic resolution [90,91], the method is suitable to understand the functional mechanism of the multi-spanning helical membrane proteins [44,92]. The multi-spanning helical membrane proteins are still a challenging target for solution NMR studies, as the apparent molecular weight of the detergent-solubilized and rHDL-embedded states often exceed 100 K. In addition, some of the proteins allow their overexpression only in insect or mammalian cells. These expression systems are not suitable for perdeuteration; thus, conventional proton detection experiments would not be practical. For those systems, low γ detection experiments, such as ^15^N-detected CRINEPT or FC-TROSY, that are less susceptible to large molecular weight even in non-deuterated conditions would be of interest [93,94,95]. Further developments of these strategies will fill the molecular weight gap between NMR and other structure methods such as X-ray and Cryo-EM and allow the establishment of the integrated structural biology for membrane proteins, in which NMR plays a pivotal role in directly connecting the dynamics of protein to the function.

## References

[1] Doyle D.A., Cabral J.M., Pfuetzner R.A., Kuo A., Gulbis J.M., Cohen S.L., Chait B.T., MacKinnon R. (1998). The Structure of the Potassium Channel: Molecular Basis of K^+^ Conduction and Selectivity. Science.

[2] Zhou Y., Morais-Cabral J.H., Kaufman A., MacKinnon R. (2001). Chemistry of ion coordination and hydration revealed by a K^+^ channel-Fab complex at 2.0 A resolution. Nature.

[3] Jiang Y., Lee A., Chen J., Cadene M., Chait B.T., MacKinnon R. (2002). Crystal structure and mechanism of a calcium-gated potassium channel. Nature.

[4] Jiang Y., Lee A., Chen J., Cadene M., Chait B.T., MacKinnon R. (2002). The open pore conformation of potassium channels. Nature.

[5] Cao E., Liao M., Cheng Y., Julius D. (2013). TRPV1 structures in distinct conformations reveal activation mechanisms. Nature.

[6] Du J., Lü W., Wu S., Cheng Y., Gouaux E. (2015). Glycine receptor mechanism elucidated by electron cryo-microscopy. Nature.

[7] Wu J., Yan Z., Li Z., Yan C., Lu S., Dong M., Yan N. (2015). Structure of the voltage-gated calcium channel Cav1.1 complex. Science.

[8] Saotome K., Murthy S.E., Kefauver J.M., Whitwam T., Patapoutian A., Ward A.B. (2018). Structure of the mechanically activated ion channel Piezo1. Nature.

[9] Zhao Q., Zhou H., Chi S., Wang Y., Wang J., Geng J., Wu K., Liu W., Zhang T., Dong M.-Q. (2018). Structure and mechanogating mechanism of the Piezo1 channel. Nature.

[10] Long S.B., Tao X., Campbell E.B., MacKinnon R. (2007). Atomic structure of a voltage-dependent K^+^ channel in a lipid membrane-like environment. Nature.

[11] Payandeh J., Scheuer T., Zheng N., Catterall W.A. (2011). The crystal structure of a voltage-gated sodium channel. Nature.

[12] McCusker E.C., Bagnéris C., Naylor C.E., Cole A.R., D’Avanzo N., Nichols C.G., Wallace B.A. (2012). Structure of a bacterial voltage-gated sodium channel pore reveals mechanisms of opening and closing. Nat. Commun.

[13] Hite R.K., MacKinnon R. (2017). Structural Titration of Slo2.2, a Na(+)-Dependent K(+) Channel. Cell.

[14] Chakrapani S., Cordero-Morales J.F., Perozo E. (2007). A Quantitative Description of KcsA Gating I: Macroscopic Currents. J. Gen. Physiol..

[15] Takeuchi K., Takahashi H., Kawano S., Shimada I. (2007). Identification and Characterization of the Slowly Exchanging pH-dependent Conformational Rearrangement in KcsA. J. Biol. Chem..

[16] Imai S., Osawa M., Takeuchi K., Shimada I. (2010). Structural basis underlying the dual gate properties of KcsA. Proc. Natl. Acad. Sci. USA.

[17] Chakrapani S., Cordero-Morales J.F., Perozo E. (2007). A Quantitative Description of KcsA Gating II: Single-Channel Currents. J. Gen. Physiol..

[18] Imai S., Osawa M., Mita K., Toyonaga S., Machiyama A., Ueda T., Takeuchi K., Oiki S., Shimada I. (2012). Functional Equilibrium of the KcsA Structure Revealed by NMR. J. Biol. Chem..

[19] Sun Z., Xu Y., Zhang D., McDermott A.E. (2020). Probing allosteric coupling in a constitutively open mutant of the ion channel KcsA using solid-state NMR. Proc. Natl. Acad. Sci. USA.

[20] Baker K.A., Tzitzilonis C., Kwiatkowski W., Choe S., Riek R. (2007). Conformational dynamics of the KcsA potassium channel governs gating properties. Nat. Struct. Mol. Biol..

[21] Bayburt T.H., Grinkova Y.V., Sligar S.G. (2002). Self-Assembly of Discoidal Phospholipid Bilayer Nanoparticles with Membrane Scaffold Proteins. Nano Lett..

[22] van der Cruijsen E.A.W., Prokofyev A.V., Pongs O., Baldus M. (2017). Probing Conformational Changes during the Gating Cycle of a Potassium Channel in Lipid Bilayers. Biophys. J..

[23] Chill J.H., Louis J.M., Baber J.L., Bax A. (2006). Measurement of 15N relaxation in the detergent-solubilized tetrameric KcsA potassium channel. J. Biomol. NMR.

[24] Chill J.H., Louis J.M., Delaglio F., Bax A. (2007). Local and global structure of the monomeric subunit of the potassium channel KcsA probed by NMR. Biochim. Biophys. Acta.

[25] Iwahashi Y., Toyama Y., Imai S., Itoh H., Osawa M., Inoue M., Shimada I. (2020). Conformational equilibrium shift underlies altered K(+) channel gating as revealed by NMR. Nat. Commun..

[26] Minato Y., Suzuki S., Hara T., Kofuku Y., Kasuya G., Fujiwara Y., Igarashi S., Suzuki E., Nureki O., Hattori M. (2016). Conductance of P2X4 purinergic receptor is determined by conformational equilibrium in the transmembrane region. Proc. Natl. Acad. Sci. USA.

[27] Mase Y., Yokogawa M., Osawa M., Shimada I. (2012). Structural basis for modulation of gating property of G protein-gated inwardly rectifying potassium ion channel (GIRK) by i/o-family G protein α subunit (Gαi/o). J. Biol. Chem..

[28] Toyama Y., Kano H., Mase Y., Yokogawa M., Osawa M., Shimada I. (2018). Structural basis for the ethanol action on G-protein-activated inwardly rectifying potassium channel 1 revealed by NMR spectroscopy. Proc. Natl. Acad. Sci. USA.

[29] Brettmann J.B., Urusova D., Tonelli M., Silva J.R., Henzler-Wildman K.A. (2015). Role of protein dynamics in ion selectivity and allosteric coupling in the NaK channel. Proc. Natl. Acad. Sci. USA.

[30] Yokogawa M., Osawa M., Takeuchi K., Mase Y., Shimada I. (2011). NMR analyses of the Gbetagamma binding and conformational rearrangements of the cytoplasmic pore of G protein-activated inwardly rectifying potassium channel 1 (GIRK1). J. Biol. Chem..

[31] Mandala V.S., Loftis A.R., Shcherbakov A.A., Pentelute B.L., Hong M. (2020). Atomic structures of closed and open influenza B M2 proton channel reveal the conduction mechanism. Nat. Struct. Mol. Biol..

[32] Hiller S., Garces R.G., Malia T.J., Orekhov V.Y., Colombini M., Wagner G. (2008). Solution Structure of the Integral Human Membrane Protein VDAC-1 in Detergent Micelles. Science.

[33] Retel J.S., Nieuwkoop A.J., Hiller M., Higman V.A., Barbet-Massin E., Stanek J., Andreas L.B., Franks W.T., van Rossum B.J., Vinothkumar K.R. (2017). Structure of outer membrane protein G in lipid bilayers. Nat. Commun..

[34] Oxenoid K., Dong Y., Cao C., Cui T., Sancak Y., Markhard A.L., Grabarek Z., Kong L., Liu Z., Ouyang B. (2016). Architecture of the mitochondrial calcium uniporter. Nature.

[35] Ge L., Villinger S., Mari S.A., Giller K., Griesinger C., Becker S., Müller D.J., Zweckstetter M. (2016). Molecular Plasticity of the Human Voltage-Dependent Anion Channel Embedded into a Membrane. Structure.

[36] Taylor K.C., Kang P.W., Hou P., Yang N.D., Kuenze G., Smith J.A., Shi J., Huang H., White K.M., Peng D. (2020). Structure and physiological function of the human KCNQ1 channel voltage sensor intermediate state. Elife.

[37] Qasim A., Sher I., Hirschhorn O., Shaked H., Qasem Z., Ruthstein S., Chill J.H. (2019). Investigation of a KcsA Cytoplasmic pH Gate in Lipoprotein Nanodiscs. ChemBioChem.

[38] Boulton S., Akimoto M., Akbarizadeh S., Melacini G. (2017). Free energy landscape remodeling of the cardiac pacemaker channel explains the molecular basis of familial sinus bradycardia. J. Biol. Chem..

[39] Ozawa S., Kimura T., Nozaki T., Harada H., Shimada I., Osawa M. (2015). Structural basis for the inhibition of voltage-dependent K+ channel by gating modifier toxin. Sci. Rep..

[40] Takeuchi K., Yokogawa M., Matsuda T., Sugai M., Kawano S., Kohno T., Nakamura H., Takahashi H., Shimada I. (2003). Structural basis of the KcsA K(+) channel and agitoxin2 pore-blocking toxin interaction by using the transferred cross-saturation method. Structure.

[41] Matsumura K., Shimomura T., Kubo Y., Oka T., Kobayashi N., Imai S., Yanase N., Akimoto M., Fukuda M., Yokogawa M. (2021). Mechanism of hERG inhibition by gating-modifier toxin, APETx1, deduced by functional characterization. BMC Mol. Cell Biol..

[42] Cao C., Wang S., Cui T., Su X.-C., Chou J.J. (2017). Ion and inhibitor binding of the double-ring ion selectivity filter of the mitochondrial calcium uniporter. Proc. Natl. Acad. Sci. USA.

[43] Shimada I., Ueda T., Kofuku Y., Eddy M.T., Wüthrich K. (2019). GPCR drug discovery: Integrating solution NMR data with crystal and cryo-EM structures. Nat. Rev. Drug Discov..

[44] Nishida N., Osawa M., Takeuchi K., Imai S., Stampoulis P., Kofuku Y., Ueda T., Shimada I. (2014). Functional dynamics of cell surface membrane proteins. J. Magn. Reson..

[45] Kofuku Y., Ueda T., Okude J., Shiraishi Y., Kondo K., Maeda M., Tsujishita H., Shimada I. (2012). Efficacy of the β₂-adrenergic receptor is determined by conformational equilibrium in the transmembrane region. Nat. Commun..

[46] Butterfoss G., DeRose E., Gabel S., Perera L., Krahn J., Mueller G., Zheng X., London R. (2010). Conformational dependence of 13C shielding and coupling constants for methionine methyl groups. J. Biomol. NMR.

[47] Nygaard R., Zou Y., Dror R.O., Mildorf T.J., Arlow D.H., Manglik A., Pan A.C., Liu C.W., Fung J.J., Bokoch M.P. (2013). The Dynamic Process of β2-Adrenergic Receptor Activation. Cell.

[48] Isogai S., Deupi X., Opitz C., Heydenreich F.M., Tsai C.-J., Brueckner F., Schertler G.F.X., Veprintsev D.B., Grzesiek S. (2016). Backbone NMR reveals allosteric signal transduction networks in the β1-adrenergic receptor. Nature.

[49] Solt A.S., Bostock M.J., Shrestha B., Kumar P., Warne T., Tate C.G., Nietlispach D. (2017). Insight into partial agonism by observing multiple equilibria for ligand-bound and Gs-mimetic nanobody-bound beta1-adrenergic receptor. Nat. Commun..

[50] Liu J.J., Horst R., Katritch V., Stevens R.C., Wüthrich K. (2012). Biased Signaling Pathways in β_2_-Adrenergic Receptor Characterized by ^19^F-NMR. Science.

[51] Manglik A., Kimm T.H., Masureel M., Altenbach C., Yang Z., Hilger D., Lerch M.T., Kobilka T.S., Thian F.S., Hubbell W.L. (2015). Structural Insights into the Dynamic Process of β2-Adrenergic Receptor Signaling. Cell.

[52] Imai S., Yokomizo T., Kofuku Y., Shiraishi Y., Ueda T., Shimada I. (2020). Structural equilibrium underlying ligand-dependent activation of β(2)-adrenoreceptor. Nat. Chem. Biol..

[53] Grahl A., Abiko L.A., Isogai S., Sharpe T., Grzesiek S. (2020). A high-resolution description of β1-adrenergic receptor functional dynamics and allosteric coupling from backbone NMR. Nat. Commun..

[54] Chung K.Y., Kim T.H., Manglik A., Alvares R., Kobilka B.K., Prosser R.S. (2012). Role of detergents in conformational exchange of a G protein-coupled receptor. J. Biol. Chem..

[55] Kim T.H., Chung K.Y., Manglik A., Hansen A.L., Dror R.O., Mildorf T.J., Shaw D.E., Kobilka B.K., Prosser R.S. (2013). The Role of Ligands on the Equilibria Between Functional States of a G Protein-Coupled Receptor. J. Am. Chem. Soc..

[56] Ye L., Neale C., Sljoka A., Lyda B., Pichugin D., Tsuchimura N., Larda S.T., Pomes R., Garcia A.E., Ernst O.P. (2018). Mechanistic insights into allosteric regulation of the A2A adenosine G protein-coupled receptor by physiological cations. Nat. Commun..

[57] Frei J.N., Broadhurst R.W., Bostock M.J., Solt A., Jones A.J.Y., Gabriel F., Tandale A., Shrestha B., Nietlispach D. (2020). Conformational plasticity of ligand-bound and ternary GPCR complexes studied by 19F NMR of the β1-adrenergic receptor. Nat. Commun..

[58] Clark L.D., Dikiy I., Chapman K., Rödström K.E.J., Aramini J., LeVine M.V., Khelashvili G., Rasmussen S.G.F., Gardner K.H., Rosenbaum D.M. (2017). Ligand modulation of sidechain dynamics in a wild-type human GPCR. eLife.

[59] O’Brien E.S., Fuglestad B., Lessen H.J., Stetz M.A., Lin D.W., Marques B.S., Gupta K., Fleming K.G., Wand A.J. (2020). Membrane Proteins Have Distinct Fast Internal Motion and Residual Conformational Entropy. Angew. Chem. Int. Ed..

[60] Kooijman L., Schuster M., Baumann C., Jurt S., Löhr F., Fürtig B., Güntert P., Zerbe O. (2020). Dynamics of Bacteriorhodopsin in the Dark-Adapted State from Solution Nuclear Magnetic Resonance Spectroscopy. Angew. Chem. Int. Ed..

[61] Kofuku Y., Ueda T., Okude J., Shiraishi Y., Kondo K., Mizumura T., Suzuki S., Shimada I. (2014). Functional dynamics of deuterated β2 -adrenergic receptor in lipid bilayers revealed by NMR spectroscopy. Angew. Chem. Int. Ed. Engl..

[62] Casiraghi M., Damian M., Lescop E., Point E., Moncoq K., Morellet N., Levy D., Marie J., Guittet E., Banères J.-L. (2016). Functional Modulation of a G Protein-Coupled Receptor Conformational Landscape in a Lipid Bilayer. J. Am. Chem. Soc..

[63] Rajagopal S., Rajagopal K., Lefkowitz R.J. (2010). Teaching old receptors new tricks: Biasing seven-transmembrane receptors. Nat. Rev. Drug Discov..

[64] Reiter E., Ahn S., Shukla A.K., Lefkowitz R.J. (2012). Molecular mechanism of β-arrestin-biased agonism at seven-transmembrane receptors. Annu. Rev. Pharm. Toxicol..

[65] Standfuss J., Edwards P.C., D’Antona A., Fransen M., Xie G., Oprian D.D., Schertler G.F. (2011). The structural basis of agonist-induced activation in constitutively active rhodopsin. Nature.

[66] Okude J., Ueda T., Kofuku Y., Sato M., Nobuyama N., Kondo K., Shiraishi Y., Mizumura T., Onishi K., Natsume M. (2015). Identification of a Conformational Equilibrium That Determines the Efficacy and Functional Selectivity of the μ-Opioid Receptor. Angew. Chem. Int. Ed..

[67] Shiraishi Y., Natsume M., Kofuku Y., Imai S., Nakata K., Mizukoshi T., Ueda T., Iwaï H., Shimada I. (2018). Phosphorylation-induced conformation of β2-adrenoceptor related to arrestin recruitment revealed by NMR. Nat. Commun..

[68] Yang F., Yu X., Liu C., Qu C.-X., Gong Z., Liu H.-D., Li F.-H., Wang H.-M., He D.-F., Yi F. (2015). Phospho-selective mechanisms of arrestin conformations and functions revealed by unnatural amino acid incorporation and 19F-NMR. Nat. Commun..

[69] Jones A.J.Y., Gabriel F., Tandale A., Nietlispach D. (2020). Structure and Dynamics of GPCRs in Lipid Membranes: Physical Principles and Experimental Approaches. Molecules.

[70] Ferré G., Eddy M.T. (2020). Structural biology of human GPCR drugs and endogenous ligands—Insights from NMR spectroscopy. Methods.

[71] Vogel A., Bosse M., Gauglitz M., Wistuba S., Schmidt P., Kaiser A., Gurevich V.V., Beck-Sickinger A.G., Hildebrand P.W., Huster D. (2020). The Dynamics of the Neuropeptide Y Receptor Type 1 Investigated by Solid-State NMR and Molecular Dynamics Simulation. Molecules.

[72] Schmidt P., Thomas L., Müller P., Scheidt H.A., Huster D. (2014). The G-protein-coupled neuropeptide Y receptor type 2 is highly dynamic in lipid membranes as revealed by solid-state NMR spectroscopy. Chemistry.

[73] Alam A., Kowal J., Broude E., Roninson I., Locher K.P. (2019). Structural insight into substrate and inhibitor discrimination by human P-glycoprotein. Science.

[74] Aller S.G., Yu J., Ward A., Weng Y., Chittaboina S., Zhuo R., Harrell P.M., Trinh Y.T., Zhang Q., Urbatsch I.L. (2009). Structure of P-Glycoprotein Reveals a Molecular Basis for Poly-Specific Drug Binding. Science.

[75] Jin M.S., Oldham M.L., Zhang Q., Chen J. (2012). Crystal structure of the multidrug transporter P-glycoprotein from Caenorhabditis elegans. Nature.

[76] Kodan A., Yamaguchi T., Nakatsu T., Sakiyama K., Hipolito C.J., Fujioka A., Hirokane R., Ikeguchi K., Watanabe B., Hiratake J. (2014). Structural basis for gating mechanisms of a eukaryotic P-glycoprotein homolog. Proc. Natl. Acad. Sci. USA.

[77] Kodan A., Yamaguchi T., Nakatsu T., Matsuoka K., Kimura Y., Ueda K., Kato H. (2019). Inward- and outward-facing X-ray crystal structures of homodimeric P-glycoprotein CmABCB1. Nat. Commun..

[78] Murakami S., Nakashima R., Yamashita E., Matsumoto T., Yamaguchi A. (2006). Crystal structures of a multidrug transporter reveal a functionally rotating mechanism. Nature.

[79] Tam H.K., Foong W.E., Oswald C., Herrmann A., Zeng H., Pos K.M. (2021). Allosteric drug transport mechanism of multidrug transporter AcrB. Nat. Commun..

[80] Du D., Wang Z., James N.R., Voss J.E., Klimont E., Ohene-Agyei T., Venter H., Chiu W., Luisi B.F. (2014). Structure of the AcrAB–TolC multidrug efflux pump. Nature.

[81] Schuldiner S. (2009). EmrE, a model for studying evolution and mechanism of ion-coupled transporters. Biochim. Et. Biophys. Acta Proteins Proteom..

[82] Penders B., Horstman K., Vos R. (2007). Proper science in moist biology. EMBO Rep..

[83] Miller C. (2007). Pretty Structures, But What About the Data?. Science.

[84] Tate C.G., Ubarretxena-Belandia I., Baldwin J.M. (2003). Conformational changes in the multidrug transporter EmrE associated with substrate binding. J. Mol. Biol..

[85] Chen Y.J., Pornillos O., Lieu S., Ma C., Chen A.P., Chang G. (2007). X-ray structure of EmrE supports dual topology model. Proc. Natl. Acad. Sci. USA.

[86] Morrison E.A., DeKoster G.T., Dutta S., Vafabakhsh R., Clarkson M.W., Bahl A., Kern D., Ha T., Henzler-Wildman K.A. (2012). Antiparallel EmrE exports drugs by exchanging between asymmetric structures. Nature.

[87] Morrison E.A., Henzler-Wildman K.A. (2014). Transported Substrate Determines Exchange Rate in the Multidrug Resistance Transporter EmrE. J. Biol. Chem..

[88] Dutta S., Morrison E.A., Henzler-Wildman K.A. (2014). Blocking dynamics of the SMR transporter EmrE impairs efflux activity. Biophys. J..

[89] Shcherbakov A.A., Hisao G., Mandala V.S., Thomas N.E., Soltani M., Salter E.A., Davis J.H., Henzler-Wildman K.A., Hong M. (2021). Structure and dynamics of the drug-bound bacterial transporter EmrE in lipid bilayers. Nat. Commun..

[90] Tokunaga Y., Viennet T., Arthanari H., Takeuchi K. (2020). Spotlight on the Ballet of Proteins: The Structural Dynamic Properties of Proteins Illuminated by Solution NMR. Int. J. Mol. Sci..

[91] Arthanari H., Takeuchi K., Dubey A., Wagner G. (2019). Emerging solution NMR methods to illuminate the structural and dynamic properties of proteins. Curr. Opin. Struct. Biol..

[92] Osawa M., Takeuchi K., Ueda T., Nishida N., Shimada I. (2012). Functional dynamics of proteins revealed by solution NMR. Curr. Opin. Struct. Biol..

[93] Takeuchi K., Arthanari H., Imai M., Wagner G., Shimada I. (2016). Nitrogen-detected TROSY yields comparable sensitivity to proton-detected TROSY for non-deuterated, large proteins under physiological salt conditions. J. Biomol. NMR.

[94] Boeszoermenyi A., Chhabra S., Dubey A., Radeva D.L., Burdzhiev N.T., Chanev C.D., Petrov O.I., Gelev V.M., Zhang M., Anklin C. (2019). Aromatic 19F-13C TROSY: A background-free approach to probe biomolecular structure, function, and dynamics. Nat. Methods.

[95] Tokunaga Y., Takeuchi K., Okude J., Ori K., Torizawa T., Shimada I. (2020). Structural Fingerprints of an Intact Monoclonal Antibody Acquired under Formulated Storage Conditions via 15N Direct Detection Nuclear Magnetic Resonance. J. Med. Chem..

